# MicroRNAs and Osteoarthritis

**DOI:** 10.3390/cells7080092

**Published:** 2018-08-01

**Authors:** Charles J. Malemud

**Affiliations:** Department of Medicine, Division of Rheumatic Diseases, University Hospitals Cleveland Medical Center, Foley Medical Building, 2061 Cornell Road, Cleveland, OH 44106-5076, USA; cjm4@cwru.edu; Tel.: +1-(216)-536-1945; Fax: +1-(216)-844-2288

**Keywords:** apoptosis, articular cartilage, autophagy, chondrocytes, extracellular matrix, microRNA

## Abstract

An imbalance in gene expressional events skewing chondrocyte anabolic and catabolic pathways toward the latter causes an aberrant turnover and loss of extracellular matrix proteins in osteoarthritic (OA) articular cartilage. Thus, catabolism results in the elevated loss of extracellular matrix proteins. There is also evidence of an increase in the frequency of chondrocyte apoptosis that compromises the capacity of articular cartilage to undergo repair. Although much of the fundamental OA studies over the past 20 years identified and characterized many genes relevant to pro-inflammatory cytokines, apoptosis, and matrix metalloproteinases (MMPs)/a disintegrin and metalloproteinase with thrombospondin motif (ADAMTS), more recent studies focused on epigenetic mechanisms and the associated role of microRNAs (miRs) in regulating gene expression in OA cartilage. Thus, several miRs were identified as regulators of chondrocyte signaling pathways, apoptosis, and proteinase gene expression. For example, the reduced expression of miR-146a was found to be coupled to reduced type II collagen (COL2) in OA cartilage, whereas MMP-13 levels were increased, suggesting an association between *MMP-13* gene expression and *COL2A1* gene expression. Results of these studies imply that microRNAs could become useful in the search for diagnostic biomarkers, as well as providing novel therapeutic targets for intervention in OA.

## 1. Introduction

The main focus of basic studies designed to unravel the pathology of osteoarthritis (OA) in humans recently employed non-surgical animal models with genetically modified mice as replicas of the human disease [1,2]. The results of these studies provided many important insights into the pathogenesis of human OA which were based mainly on compelling evidence that chondrocyte metabolic pathways that regulate anabolic and catabolic events were skewed toward the latter [3]. Thus, at its most fundamental level, human OA is characterized by chronic inflammation, progressive destruction of articular cartilage, and subchondral bone sclerosis [4]. 

In addition to the significance of molecular events that drive the progression of OA, there is now ample evidence that “chondroscenesence”, a term used to describe chondrocytes in the older individual, was linked to inflammation and was characterized by an abnormal interplay between autophagy and the inflammasome [5]. Of note, it was also previously postulated using a comparison-type analysis that OA-like cartilage lesions in “elderly” rats responded more favorably to moderate physical activity and normal mechanical loading compared with rats not subjected to the exercise program [6]. This suggested that physical activity could potentially blunt the progression of OA even in older individuals, which may be associated with improved lubrication of the articular cartilage.

Recent advances also increased our understanding of how the loss of articular cartilage extracellular matrix (ECM) proteins characteristic of OA synovial joints results from the action of pro-inflammatory cytokines (e.g., interleukin-1β (IL-1β), IL-6, IL-15, IL-17, IL-21) and several other interleukin family members [7,8,9]. In addition to several of these interleukins, tumor necrosis factor-α (TNF-α) mediates the upregulation of matrix metalloproteinases (MMPs), and elevated levels of a family of enzymes termed a disintegrin and metalloproteinase with thrombospondin motif (e.g., ADAMTS-4,-5) with selective aggrecanase (i.e., aggrecanase-1 and aggrecanase-2) activities [10,11,12]. Additional soluble mediators of inflammation, including nitric oxide and the transcription nuclear factor kappa b (NF-κB), as well as aberrant mechanical stressors, also appear to be involved in cartilage ECM protein degradation in OA [4]. However, the medical therapy of OA remains fixated on primarily relieving symptomatic synovial joint pain [12]. This appears to be in keeping with the identification of various pain pathways which are implicated in the progression of OA [13,14,15].

Basic studies also implicated programmed cell death, also known as apoptosis, or in the chondrocyte context, “chondropotosis”, in the OA pathological process [16]. The increased frequency of apoptotic chondrocytes in human OA leads to reduced chondrocyte vitality, which is accompanied by a feeble response of articular cartilage for repairing OA lesions [17,18,19]. In this context, Terminal deoxynucleotidyl transferase dUTP nick end labeling.

(TUNEL)/Hoechst 33258 staining revealed an elevated frequency of apoptosis in freshly isolated chondrocytes from OA cartilage [20], as well as altered patterns of pro-apoptosis and anti-apoptosis factors, exemplified by an analysis of the extrinsic apoptosis pathway which was determined by measurements of Bcl-2-associated X protein( Bax), B-cell lymphoma-2 (Bcl-2), TNF-related apoptosis-inducing ligand (TRAIL), death receptor-5 (DR5), and caspase-3 [21].

In addition to those genes which effectively reduce chondrocyte viability, other specific genes and signaling pathways, include genes that encode bone morphogenetic proteins, the WNT/β-catenin proteins, leukemia inhibitory factor, hypoxia-inducible factor-1-α/2α (HIF-1α/2α) [22,23], as well as *GREM1*, *FRZB*, *DKK1*, *VEGF*, *EGF* [23,24,25], *GDF5* [26] genes, and the NOTCH/NF-κB pathway [27]. Each of these were linked to various stages in the OA cartilage degenerative process. 

Significant advances also occurred in defining the role that signal transduction pathways play in OA. For example, pro-inflammatory cytokine-mediated activation of mitogen-activated protein kinase (MAPK) and Janus kinase/signal transducers and activators of transcription (JAK-STAT) pathways demonstrated how these signaling pathways control gene expression associated with pro-apoptosis protein synthesis and MMP synthesis, both of which were found to be dysregulated in OA articular chondrocytes [28,29,30,31,32,33].

## 2. A Role for Epigenetics in OA

Epigenetics is a field of genetics wherein variations in cell phenotype are considered to result from external or environmental factors that affect how gene activity is regulated. This mechanism of gene regulation exists in contradistinction to genes regulated by changes in DNA sequence. Thus, in this way, epigenetic factors can regulate the dynamic potential of gene transcription.

Results of recent studies implicated epigenetics as an important contributor to the pathology and progression of cartilage ECM protein turnover and damage in OA. In that regard, genome-wide association studies (GWAS) identified several critical genes, including *IL-1β*, *IL-6*, and *TNF-α*, which were subject to epigenetic modifications during the OA process [34]. In fact, these genes and others were implicated in inflammatory processes characteristic of OA progression, as well as how these genes could be responsible for producing the chondrocyte hypertrophic phenotype characteristic of altered cartilage structure in OA [35]. Of note, the repertoire of altered methylation patterns in fibroblast-like synoviocytes that defines the inflammatory process in rheumatoid arthritis (RA) was employed to distinguish primary inflammatory episodes in RA from those found in OA synovium. Furthermore, an integrative analysis of the methylome transcriptome and proteome from OA chondrocytes defined several components which could be amenable to therapeutic intervention [36]. In addition, an epigenetic analysis shed new light on the role of methylation in regulating the gene expression of transcription factors, cytokines, ECM proteins, and MMPs pertinent to OA [37]. Just as critical to this analysis were the results stemming from a review of GWAS in OA [38]. This review concluded that there were several types of factors that contributed to the development of OA [38]. Thus, GWAS deemed systemic and genetic factors (e.g., *GDF5*, *ASTN2*, *DOT1L* genes), intrinsic factors (e.g., congenital defects, past surgeries, infection), as well as extrinsic biomechanical factors (e.g., physical activity, high body mass index (BMI), past joint injury) as particularly germane to the development of OA. 

## 3. MicroRNAs and OA

### Overview

Microribonucleic acids (miRNAs; miRs) are ~22-nucleotide non-coding RNAs which interact with cognate messenger RNAs (mRNAs) [39]. Thus, it appears that the principal role of miRs is to modify posttranscriptional regulation of genes either through enhancement of degradation, by suppressing translation, or via other mechanisms. 

Several groups of investigators [40,41,42,43,44] proposed that miRs could play a key role in OA by virtue of the fact that mechanical loading was shown to affect miR expression. In support of this contention, previously published evidence indicated that miRs were regulators of hundreds of genes which were relevant to cartilage development, homeostasis, and OA pathology [45]. The results of many studies reviewed by Wu et al. [46] revealed more than 25 miRs which were implicated in cartilage development and OA, and, in particular, miRs which were associated with regulating chondrocyte hypertrophy, and proteolytic enzyme synthesis. Of note, OA cartilage signaling pathways, exemplified by those involving transforming growth factor-ß (TGF-β)/SMA and Drosophila MADs (SMADs)/Bone Morphogenetic Proteins (BMPs), MMPs, ADAMTS, inducible nitric acid synthase (iNOS) IL-1, and TNF-α, were found, in part, to be regulated by miRs [47]. Furthermore, general pathologic phenomena associated with cartilage degeneration and OA, including inflammation, obesity, apoptosis, and defective autophagy were also found to be related to the specific activity of miRs [19]. With respect to autophagy, miRs were reported to most likely to play an important role in the aberrant autophagic response of OA chondrocytes by virtue of their capacity to regulate apoptosis [48,49] and reactive oxygen species [49]. 

Specifically, several miRs were identified as playing a role in OA pathology by virtue of their abnormal expression in OA. These included miR-9, miR-27, miR-34a, miR-140, miR-146a, miR-558, and miR-602 [50]. In fact, some of these miRs were identified as regulators of interleukin-mediated expression of MMP-13 (i.e., collagenase-3), an MMP critical in the degradation of cartilage type II collagen. Of note, Cong et al. [50], in reviewing the published literature, indicated that many miRs were differentially expressed in OA, whereby upregulated miRs primarily targeted events occurring in the nucleus, and downregulated miRs primarily targeted transcription. 

## 4. Specific miRs: Role in Chondrocyte Signaling Pathways, Apoptosis, and Proteinase Production

### 4.1. Specific miRs: Chondrocyte Signaling

One approach to recognizing the extent to which miRs may be involved in altering articular cartilage homeostasis was conducted by determining which miRs alter various aspects of chondrocyte signaling, apoptosis, and proteinase production [51]. In that regard, the results of several recent studies focused on how miRs regulate those events which are germane to OA pathophysiology. For example, Zhang et al. [52] showed that miR-210 targeted the 3′-untranslated region (UTR) of death receptor-6 (DR6) in cultured chondrocytes which resulted in the inhibition of DR6 gene expression. The inhibition of DR6, in turn, inhibited NF-κB-mediated signaling. A similar finding was reported in vivo in rats using the anterior cruciate ligament transection model of OA whereby mIR-210 expression was also reduced. Of note, cytokine production, and NF-κB and DR6 expression were all inhibited following miR-210 lentivirus administration to the animals, suggesting that miR-210 was involved in altering articular cartilage homeostasis in this model of experimental OA. However, miR-210 is not the only miR involved in NF-κB pathway signaling; miR-26a and miR-26b suppressed karyopherin subunit alpha-3 (*KPNA3*) gene expression, the latter identified as a mediator affecting the capacity of the NF-κB p65 subunit to translocate from the cytoplasm to the nucleus [45]. Importantly, levels of MMP-3, -9, and -13, as well as of cyclooxygenase-2 (COX-2), were upregulated after chondrocytes were transfected with an inhibitor of miR-26a or miR-26b [53]. In addition, the levels of miR-26a/miR-26b, as well as of miR-138 and miR-140, were reduced in cartilage from OA patients [53]. Of note, Wei et al. [51] also showed that miR-138 was greatly reduced in OA cartilage compared to normal cartilage. Moreover, miR-138 was reduced following incubation of both OA and normal human chondrocytes with TNF-α, whereas miR-138 overexpression suppressed p65, COX-2, and IL-6 in human OA chondrocytes and in the SW1353 line of chondrogenic cells. Additional miRs were also implicated in altering chondrocyte signaling pathways and gene targets (Table 1). 

Most of the targets shown in Table 1 were cited from evidence presented in papers from the PubMed database (https://www.ncbi.nlm.nih.gov/pubmed). This evidence demonstrated that many of the miRs (e.g., miR-29, miR-130a, miR-138, miR-139, miR-210, miR-26a, miR-26b, miR-140, miR-130a, and miR-27a–3p) were implicated in targeting those genes that are involved in dysregulated signal transduction (e.g., *MAPK, IGFR1*, *PI3K*, *SMAD1*, *NF-κB*), ECM protein turnover (e.g., *COL2A1, COL1A1*), pro-inflammatory cytokines (e.g., *TNFα*, *IL1ß*, *IL6*), methylation/demethylation (e.g., histone deacetylase-2, -4), MMP and ADAMTS (e.g., *MMP13*, *TIMP1*, *ADAMTS5*), and autophagy (e.g., *Atg3*, *Atg5*, *Atg14*), many of which were shown to be critical in altering the chondrocyte phenotype characteristic of OA cartilage [51,52,53,54,55,56,57,58,59].

### 4.2. Specific miRs: Apoptosis

Apoptosis is regulated by the balanced expression of pro-apoptotic and anti-apoptotic proteins, including the influential effects of a class of proteins termed cellular inhibitor of apoptosis proteins (c-IAPs) [32]. Evidence from animal models of OA and from measurements in human OA cartilage showed that the frequency of apoptotic chondrocytes was significantly increased in human OA cartilage [4,17,19]. The results of a study indicated a relationship between miR-146a and additional biomarkers of inflammation, including mothers against decapentaplegic homolog 4 (SMAD4) and vascular endothelial growth factor (VEGF) [74]. Importantly, upregulation of miR-146a increased apoptosis in human chondrocytes in association with increased levels of *VEGF* and decreased *SMAD4* gene expression. Consequently, downregulation of miR-146a reduced human chondrocyte apoptosis, whereas upregulation of VEGF induced by miR-146a was shown to correlate with SMAD4 in chondrocytes stimulated by mechanical injury [75]. More recent studies also indicated that over-expression of miR-34a induced chondrocyte apoptosis while also inhibiting proliferation which involved sirtuin-1 (SIRT1)/p53 signaling. Thus, an anti-miR-34a sequence administered to rats who had developed OA-like cartilage lesions following anterior cruciate ligament transection reduced the progression of cartilage degradation in this rodent OA model [76]. Wu et al. [77] showed that miR-181, which targets phosphatase and tensin homolog deleted on chromosome 10 (PTEN), a regulator of the phosphatidylinositol 3-kinase (PI3K)/Akt/Mechanistic Target of Rapamycin (mTOR) signaling pathway [78], upregulated the expression of caspase-3, poly-ADP-ribose polymerase (PARP), MMP-2, and MMP-9. These changes inhibited chondrocyte proliferation while also inducing apoptosis. Taken together, the results of these experimental studies indicated that targeting genes with miRs could ultimately become an effective way of thwarting the elevated frequency of apoptotic chondrocytes characteristic of human cartilage in OA synovial joints. 

### 4.3. Specific miRs: MMPs and ADAMTS

A host of miRs were discovered to regulate the expression of several MMPs and ADAMTS which are relevant to the regulation of chondrocyte homeostasis, and to the pathogenesis and progression of OA. Thus, it was instructive in establishing a role for miRs in altering human OA cartilage ECM when Yamasaki et al. [74] showed that expression of miR-146a and *COL2A1* gene expression (type II collagen) were decreased in OA cartilage with low-grade OA, whereas MMP-13 expression was increased at a similar cartilage grade, suggesting that decreased miR-146a was associated with elevated MMP-13 levels. The results of this study [74] also showed that miR-146a increased following the incubation of human chondrocytes with IL-1β. In another study, Song et al. [79] showed that miR-21 regulated the expression of the growth arrest-specific 5 gene (*GAS5*) in normal and OA regions of human cartilage removed at total knee replacement. In addition, overexpression of *GAS5* increased the expression of MMPs, including *MMP2*, *MMP3*, *MMP9*, *MMP13*, and *ADAMTS4*, while also stimulating apoptosis and suppressing autophagy. Furthermore, miR-21 was also shown to regulate GAS5, as evidenced by its reduced level in OA and the capacity of miR-21 to significantly increase cartilage destruction in “OA mice”. Song et al. [80] also showed that miR-222 regulated MMP-13 via *histone deacetylase 4* (*HDAC4*). Thus, miR-222 was significantly reduced in OA chondrocytes, and overexpression of miR-222 suppressed apoptosis while also reducing *HDAC4* and *MMP13* gene expression. The biomarker of senescence, also known as p161INK4a, was shown to be a target for miR-24 [81], and when *p161INK4a* was over-expressed in chondrocytes, the levels of *MMP1* and *MMP13* increased. Thus, miR-24 was identified as a negative regulator of p161INK4a, as well as of cartilage development and OA. A few years ago, Meng et al. [82] also identified miR-320 as a regulator of *MMP13*, where NF-κB and MAPK activation was shown to downregulate miR-320 expression. This conclusion was later extended by Jin et al. [83], who showed that overexpression of miR-320a, which targets the pre-B-cell leukemia transcription factor 3 (*PBX3*) gene, increased MMP-13, whereas *PBX3* and the proteasome inhibitor, MG132, suppressed the effects of miR-320a on the expression of *COL2A1*, *ACAN* (i.e., the aggrecan gene), sulfated glycosaminoglycans, and MMP-13. Moreover, miR-105 was also found to be important for the fibroblast growth factor-2 (FGF2)-induced gene expression of *ADAMTS4*, *5*, *7*, and *12* [84]. These are enzymes with activity toward type II collagen and aggrecan in OA. In addition, miR-105 was reduced in OA and was inversely correlated with *Runx2* expression. Thus, the FGF2/p65/miR-105/ADAMTS axis appears to be important in OA pathogenesis. Mao et al. [85] also reported that miR-92a–3p regulated *ADAMTS4/ADAMTS-5* expression, which also involved NF-κB and MAPK activation. Additionally, miR-30a was also identified as a regulator of ADAMTS-5 [86], whereby IL-1β suppressed miR-30a expression via activation of the activator protein-1 (AP-1) complex consisting of c-jun/c-fos. Thus, AP1/miR-30a appears to be essential for IL-1β-induced increase in *ADAMTS5* gene expression. 

## 5. Conclusions and Future Perspectives

Although persuasive evidence confirmed the critical role of pro-inflammatory cytokines as potent inducers of chronic inflammation, apoptosis, and proteinase gene expression in OA, the results of recent studies extended this analysis to demonstrate how various miRs regulate the effects of these cytokines, the balance between pro-apoptotic and anti-apoptotic proteins [87], and *MMP/ADAMTS* gene expression [88], all of which contribute to altered articular cartilage structure in OA. However, the complexity inherent in these responses, which resulted from identification of the numerous miRs involved in the pathogenesis and progression of human OA, created uncertainty as to how employing exogenous miRs could alter articular cartilage ECM aberrant turnover and ECM degradation, all of which are consistent characteristics of OA progression to joint failure. Perhaps more germane to their potential as translational medicines is that miRs were employed to identify additional targets for intervention in OA, including those involved in autophagy and proteasome-mediated protein degradation [89]. Furthermore, results of experimental studies performed in well-validated animal models of OA in which miRs were administered to these animals confirmed that miRs were active in vivo *T*. Thus, the results of these pre-clinical studies provide a compelling platform for considering the use of miRs in OA clinical safety trials going forward. 

## Figures and Tables

**Table 1:** Chondrocyte signaling pathways and genes regulated by microRNAs (miRs).

miR	Signaling Target/Other Targets	Reference
miR-139	*IGF1R*^1^/*EIF4G2*^2^	[54]
miR-140	*SMAD1* ^3^	[55]
miR-29a/miR-140	IL-1β/*MMP13* ^4^ ↓/*TIMP1* ^5^ ↑	[56]
miR-130a	*Tumor necrosis factor-α* ↓	[57]
miR-27a–3p	MAPK; NF-κB/*ADAMTS5* ^6^	[58]
	*SMAD3* ^7^	[59]
miR-634	*PI3K*^8^/*PIK3R1*^9^	[60]
miR-449a	IL-1β/*SIRT1* ^10^	[61]
miR-9	L-1β/IL-6; (*MCP1*) ^11^	[62]
miR-29	SMAD; NF-κB; WNT-related genes, *FZD3* ^12^, *FZD5* ^13^, *DVL3* ^14^, *FRAT2* ^15^, *CK2A2* ^16^	[63]
miR-92a-3p	*Histone deacetylase 2*↓	[64]
miR-381	*Histone deacetylase 4*↓	[65]
miR-370/miR-373	*SHMT2*^17^/*MECP2*^18^	[66]
miR-21	*GDF5* ^19^	[67]
miR-29b	*COL2A1*^20^↓; *COL1A1*^21^ ↑	[68]
miR-146a	*Camk2d*^22^/*Ppp32r*^23^	[69]
miR-146b	*SOX5* ^24^	[70]
miR-155	Autophagy-Related Genes: *Ulk1*; *FoxO3*; *Atg14*; *Atg5*; *Atg3*; *Gabarappl1*; *Map1lc3*	[71]
miR-30b	*BECN1*^25^/*Atg5*	[72,73]

^1^ Insulin-like growth factor-1 receptor; ^2^ eukaryotic translation initiation factor 4 gamma 2; ^3^ mothers against decapentaplegic homolog 1; ^4^ Matrix Metalloproteinase-13; ^5^ Tissue Inhibitor of Metalloproteinase-1. ^6^ a disintegrin and metalloproteinase with thrombospondin motif-5; ^7^ mothers against decapentaplegic homolog 3; ^8^ phosphatidylinositol 3-kinase; ^9^ regulatory subunit 1 of class 1 PI3K (p85α); ^10^ NAD-dependent deacetylase sirtuin-1; ^11^ monocyte chemoattractant protein-induced protein 1; ^12^ frizzled class receptor 3; ^13^ frizzled class receptor 5; ^14^ disheveled segment polarity protein 3; ^15^ frequently rearranged in advanced T-cell lymphomas 2; ^16^ *CK2A2* encodes a subunit of protein kinase CK2; ^17^ serine hydroxymethyl transferase-2; ^18^ methyl Cpg binding protein 2; ^19^ growth/differentiation factor 5; ^20^ Collagen Type II; ^21^ Collagen Type I; ^22^ calcium/calmodulin-dependent protein kinase II delta; ^23^ phosphatase 2 regulatory subunit B; ^24^ SRY-related HMG-box; ^25^ beclin-1.
